# Evaluation of the Surrounding Ring of Two Different Extra-Short Implant Designs in Crestal Bone Maintanence: A Histologic Study in Dogs

**DOI:** 10.3390/ma11091630

**Published:** 2018-09-06

**Authors:** José Luis Calvo-Guirado, Hilde Morales-Meléndez, Carlos Pérez-Albacete Martínez, David Morales-Schwarz, Roni Kolerman, Manuel Fernández-Domínguez, Sérgio Alexandre Gehrke, José Eduardo Maté-Sánchez de Val

**Affiliations:** 1 Faculty of Health Sciences, Universidad Católica San Antonio de Murcia, 30107 Murcia, Spain; cperezalbacete@ucam.edu (C.P.-A.M.); jemate@ucam.edu (J.E.M.-S.d.V.); 2 International Dentistry Research Cathedra Faculty of Health Sciences, Universidad Católica San Antonio de Murcia, 30107 Murcia, Spain; hildemmoralea90@gmail.com; 3 Private practice, Calle Estadío nº1, 47006 Valladolid, Spain; dgms65@gmail.com; 4 Department of Periodontology and Dental Implantology, the Maurice and Gabriela Goldschkeger School of dental Medicine, Tel Aviv University, 6934203 Tel Aviv, Israel; daniaron@netvision.net.il; 5 Faculty of Dentistry, Department of Oral and Implant Dentistry, Universidad San Pablo CEU, Grupo HM (Hospital Madrid), 11600 Madrid, Spain; clinferfun@yahoo.es; 6 Biotecnos-Tecnologia e Ciencia Ltda, 11100 Montevideo, Uruguay; sergio.gehrke@hotmail.com

**Keywords:** extrashort dental implants, implant survival, marginal bone loss, dogs experiment, wide ring, narrow ring

## Abstract

The aim of this study was to compare the implant stability and bone resorption and formation of two different extra-short implant designs with different diameter rings placed in a dog´s maxilla. Thirty-six extra-short, 5 mm diameter × 4 mm length (Short DM^®^, Bioner Sistemas Implantológicos, Barcelona, Spain), delayed implants were placed in each hemimaxilla of six dogs at the bone crest level. Eighteen implants of each design (wide and narrow ring) were installed. After 8 and 12 weeks of healing, histomorphometric analyses of the specimens were carried out to measure the crestal bone level values and the tissue thickness around the wide and narrow ring implant designs. In the microscopic analysis, less buccal bone resorption was observed in the narrow ring implants with a statistical significance (*p* < 0.001). For the peri-implant tissue thickness, the distance from the implant shoulder to the external portion of the epithelium was significantly higher for the implants installed with a wide ring with statistical significance (*p* < 0.001). Our findings suggest that the amount of peri-implant tissues (crestal bone loss) after remodeling over a period of 12 weeks was smaller in the narrow ring extra-short implant installed in the healed maxilla, compared with the wide ring extra-short implants.

## 1. Introduction

At the atrophic jaw, the amount of cortical bone remains stable, while most of the resorption occurs at the expense of the cancellous bone [1,2]. The maxillary sinus and the inferior alveolar nerve in the posterior maxilla and mandible limits, in many cases, the availability of the bone to place standard implants [3,4]. To solve these cases, several surgical techniques have been proposed, namely: guide bone regeneration, sinus lift, bone distraction, alveolar nerve transposition, angled implants, zygomatic and pterygoid, and short implants, among others [5,6,7]. Although there is a high success rate with these methods, several drawbacks are associated with these procedures, such as a high morbidity, increase in cost, more surgical procedures, and the appearance of post-operative complications after these methods, such as nerve paresthesia, sinusitis, bone graft exposure, swelling, pain, among others [8,9,10,11,12,13].

Many definitions have been proposed for short implants as well as for extra-short implants. It is accepted nowadays that short implants are those that are less than 8 mm [14].

Short implants (less than 8 mm) have been proposed as a less invasive alternative to treat the posterior atrophic jaws [8,9,10,11,12,13]. Some authors used extra-short implants in the atrophic maxilla with Guided Bone Regeneration (GBR), and suggest that short implants may be a cheaper and faster treatment compared with the longer implants in the augmented atrophic maxillary bone [15].

Short implants present the advantage of being less traumatic and are proposed as the treatment of choice for reducing the processing time, cost, and morbidity for the patient [16,17,18,19].

The survival, success, and bone loss rate of the short implants (≤8.5 mm long) was 90% in all of the groups at the three year follow-up. It seems that the design of the implant can influence the behavior of the peri-implant bone at the crestal level [20].

Extra-short implants are considered those that are less than 5 mm in length (Slotte et al., 2012) [21]. Short implants present long-term success rates, comparable to standard implants. Although many short implants present an unfavorable crown to implant ratio, they present a high success rate, comparable to standard implants [21,22]. There have been numerous studies focused on the biomechanics of short implants. In these previous studies, it has been concluded that higher rates of bone stress occur independently of the length of the implants, and there is a greater involvement of the implant diameter [23]. Also, it has been reported by previous studies that the width of the implants has more influence on the osseointegration and survival rate than the presence of additional length.

In these implants, because of their small contact surface with the bone compared with normal implants, a macro and micro design is a crucial aspect to be considered [24]. 

The development of new surface treatments increases the surface area of the implant, allowing for more bone to implant contact. Most works still favour the surface treatment of dental implants producing good substrate surfaces for osseointegration, with a great surface roughness. The reduction of the total length of the implant is because it increases the bone–implant contact due to surface roughness [25,26,27,28].

Calvo-Guirado et al. showed that extra-short implants can support individual fixed bridges and overdentures in patients with posterior bone resorption with narrow ridges [29].

Some studies describe the tendency of short implants to have a high failure rate during the first year [30]. Its proposed that this occurs as a result of the lower primary stability, because of less bone contact during the healing period [16].

In a short implant, most of the primary stability lies on the cortical bone. Therefore, adding a ring to the cervical area of a short implant design increases the contact area and support with the dense cortical bone.

The aim of this study is to evaluate the crestal bone resorption around two different extra-short implant designs in animals.

## 2. Materials and Methods

This was an experimental study that was conducted in the animal facilities at Murcia University. The manuscript was prepared following the ARRIVE (Animal Research: Reporting of In Vivo Experiments) guidelines.

Six Beagle dogs, of approximately one, to one and half years of age, were used in this study. The Ethics Committee for Animal Research at the University of Murcia (Spain) approved the study protocol, which followed the guidelines established by the European Union Council Directive of February 2013 (R.D.53/2013). The number of the procedure was A1320141102 (Animal Health Service, Murcia, Spain). 

In the clinical examination, all of the animals had a good general health; their maxillas’ were all intact, with minimal resorption and without major oral lesions.

The animals were given vaccines and vitamins against rabies, and were then put in quarantine. The dogs were kept in individual cages throughout the project and they also received adequate veterinary care. After each surgery (two procedures), the animals received an antibiotic of 6 mg/kg Clindamycin (Clindaseptin 75 mg, Chanelle Pharmaceuticals, 20 Ireland) twice daily, and an anti-inflammatory of 0.30 mg/kg Caprox Vet 100 mg (Vibrac, Spain) three times per day, systemically.

### 2.1. Surgical Procedure

The animals were pre-anaesthetized with acepromazine (0.12–0.25% mg/kg), buprenorphine (0.01 mg/kg), and medetomidine (35 μg/kg). The mixture was injected intramuscularly in the femoral quadriceps. The animals were then taken to the operating theater where, at the earliest opportunity, an intravenous catheter was inserted (diameter 22 or 20 G) into the cephalic vein, and propofol was infused at a rate of 0.4 mg/kg/min, at a slow constant infusion rate. The conventional dental infiltration anesthesia (articaine 40 mg, 1% epinephrine) was administered at the surgical sites. These procedures were carried out under the supervision of a veterinary surgeon. Maxilary premolar extractions (P2, P3, and P4) were performed bilaterally. After two months of healing, the crestal incisions were performed bilaterally in the premolar region of the maxilla. The full-thickness mucoperiosteal flaps were elevated, and the recipient sites in the premolar regions on both sides of the maxilla were prepared for the present experiment, while the other regions were used for different experimental purposes, the results of which are reported elsewhere. The healed bones were prepared in order to place extra-short implants with two different types of rings. The tested implant was a tissue level implant with a 1.9 mm smooth neck, therefore leaving space for the biological width and for reducing the marginal bone loss; this helps us measure the marginal bone reaction to the tested ring device.

**Figure 1:**
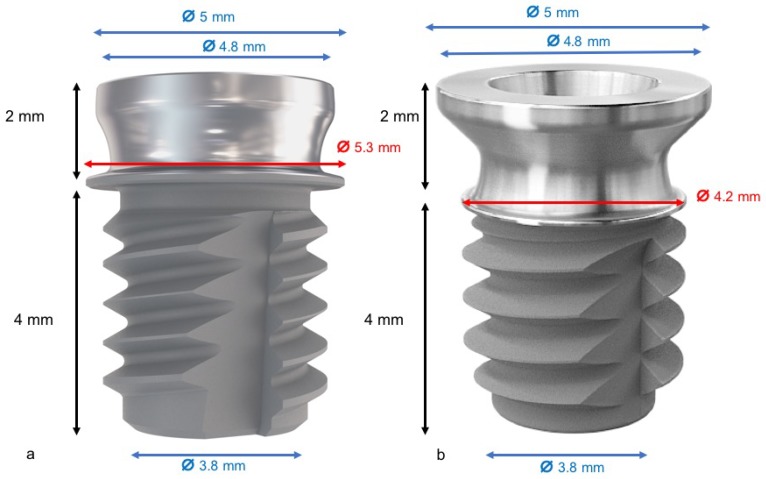
(**a**) Extra-short implant with wide cervical ring with a 5.3 mm diameter; (**b**) extra-short implant with a narrow cervical ring with a 4.2 mm diameter.

Thirty-six implants, Short DM^®^ (Bioner, Sistemas Implantológicos, Barcelona, Spain), of 4 mm in length with a 5 mm diameter, were placed. One implant was used with a narrow cervical ring with a 4.2 mm diameter, and the other was a wide cervical ring with a 5.3 mm diameter (Figure 1).

**Figure 2:**
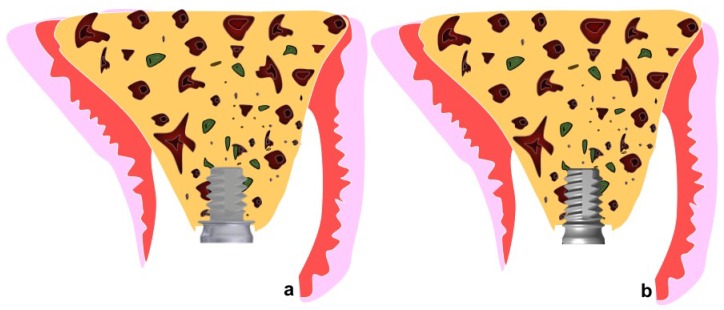
(**a**) Wide ring extra-short implant after flap elevation; (**b**) narrow ring extra-short implants installed in the maxilla.

**Figure 3:**
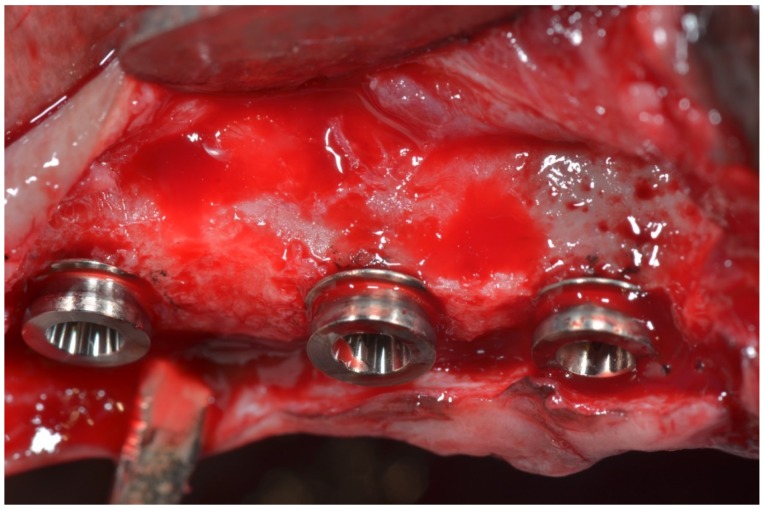
Clinical approach of wide and narrow ring extra-short implants installed in maxilla.

According to the ARRIVE, the information about the allocation/randomization must to be provided. Information about the allocation/randomization of a total of 36 implants were randomly installed. Eighteen extra-short dental implants, six per dog, with wide diameter ring (5.3 mm), and 18 with a narrow diameter ring (4.2 mm), were installed in the healed maxillas (Figure 2 and Figure 3).

The flaps were sutured with silk 4.0 (Lorca Marin, Lorca Murcia, Spain). After the surgical procedures, the animals received antibiotic treatment (Amoxicillin 500 mg, twice a day) and analgesics (ibuprofen 600 mg, three times a day), systemically. In addition, the dogs were fed a soft diet for seven days, and plaque control was maintained by the application of Sea4 Encías^®^ (Blue Sea Laboratories, Alicante, Spain). The wounds were inspected daily for postoperative clinical complications. Two weeks after surgery, the sutures were removed.

### 2.2. Histological and Histomorphometric Analysis

**Figure 4:**
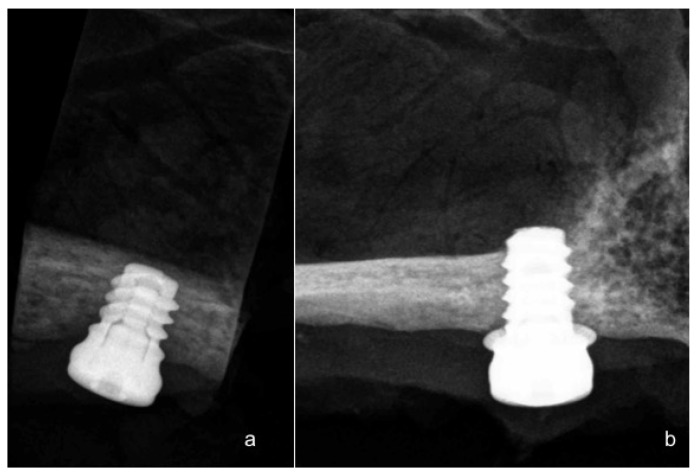
(**a**) Narrow ring extra-short implant radiograph after 90 days of evaluation; (**b**) Wide ring extra-short implant radiograph after 90 days follow-up.

Three of the animals were sacrificed after 8 weeks, and the other three animals were sacrificed after 12 weeks, after the insertion of the implant, through an overdose of Pentothal Natrium^®^ (Laboratorios Abbot, Madrid, Spain), and were perfused through the carotid arteries with a fixative containing 5% glutaraldehyde and 5% formaldehyde. Radiographs were taken after sacrifice at 60 days for the first three dogs, and at 90 days for the remaining three (Figure 4).

**Figure 5:**
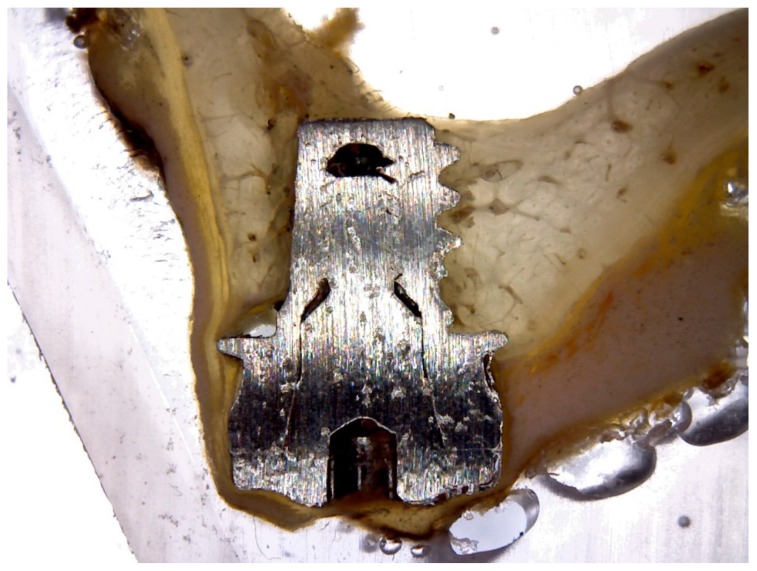
After polymerization, the specimens were sectioned along their longitudinal axis with a high precision diamond disk, at about 150 to 30 μm.

The specimens were washed in saline and were fixed in a 10% buffered formalin. The specimens were processed to obtain a thin section of soil with the automated system Precise 1 (Assing, Rome, Italy). The specimens were dehydrated in ascending series with alcohol, and were embedded in a glycol methacrylate resin (Technovit 7200 VLC, Kulzer, Wehrheim, Germany). After polymerization, the specimens were sectioned along their longitudinal axis with a high precision diamond disk, at about 150 to 30 μm. A total of two slides were obtained for each implant (Figure 5). 

The slides were stained with toluidine blue and were observed under a normal transmitted light microscope and a polarized light microscope (Leitz, Wetzlar, Germany).

**Figure 6:**
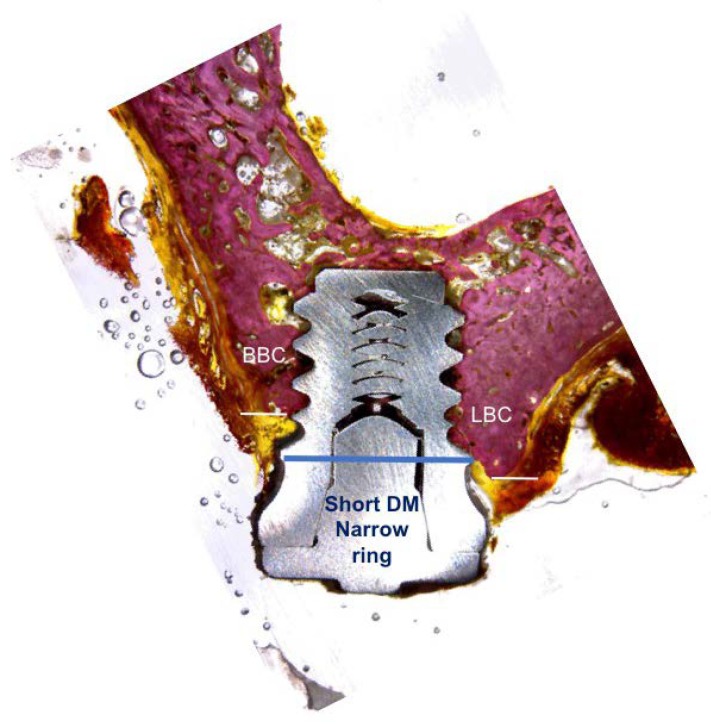
Narrow ring extra-short implant.

**Figure 7:**
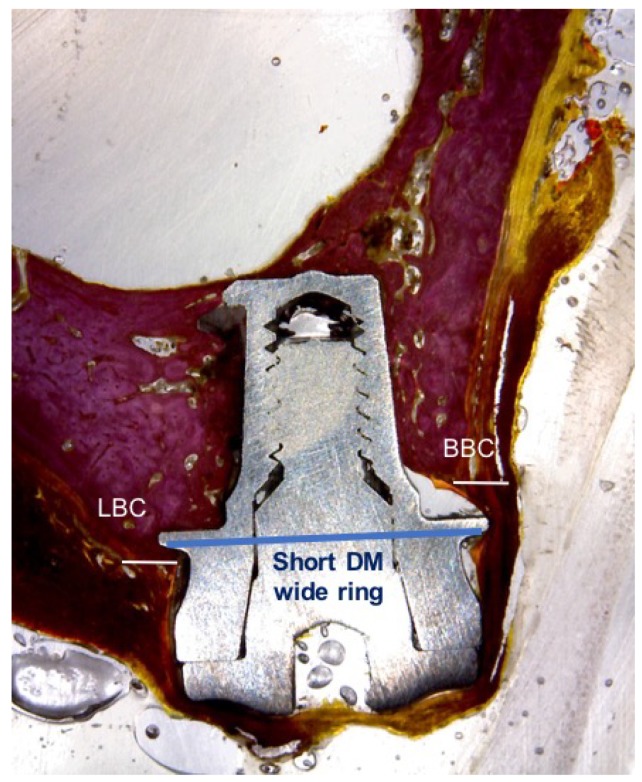
Wide ring extra-short implant.

The histological preparation evaluates the distance from the top of the implant collar to the first contact of the buccal and lingual bone (BBC and LBC), as well as the heights of the buccal and lingual bone ridges, with respect to the neck of the implant (Figure 6 and Figure 7). The resorption of the buccal bone wall compared to the reabsorption of the lingual bone wall was expressed as a linear measure. 

The buccal and lingual bone plates were measured from the implant shoulder to the first Bone to implant Contact (BIC) and to the top of the bony crest. The percentage of BIC of the native bone was also measured along the perimeter of the implant, between the coronal end of the osseointegration in the buccal and lingual aspects. The apical portion of each implant was excluded from the measurement. The total amount of bone in contact with the implants was calculated as the sum of the native bone and the newly formed bone (BIC%). A histomorphometry of the BIC percentages was performed using a light microscope (Laborlux S, Leitz) connected to a high-resolution video camera (3CCD, JVC KY-F55B, JVC^®^, JVC, Yokohama, Japan), and interconnected to a monitor and Personal Computer (Intel Pentium III 1200 MMX, Intel^®^, Intel, Santa Clara, CA, USA). This optical system was associated with a scanning pad (Matrix Vision GmbH, Oppenweiler, Germany) and a software package for histometry, with image capturing capabilities (Image-Pro Plus 4.5, Media Cybernetics Inc., Immagini and Computer Snc, Milano, Italy). The total amount of bone in contact with the implants was calculated as the sum of the native bone and the newly formed bone.

### 2.3. Statistic Analysis

The data were compared using the one-way ANOVA (Analysis of Variance) statistical tests (α = 5%), because we had two different periods of time for the evaluation (8 and 12 weeks) and two different types of implants.

The mean values and standard deviations were calculated using a BIC descriptive test and the bone resorption measurements. The values were recorded as the mean ± standard deviation. The Wilcoxon test was applied to the comparison of the mean averages and to quantify the relationships between the differences, with a 95% interval of confidence. The Bruner and Langer non-parametric was applied to the mean values for the crestal and subcrestal implants. All of the histomorphometric parameters were analyzed using descriptive methods (SPSS 19.0, SPSS, Chicago, IL, USA). For all of the tests performed, the significance level chosen was 5% (*p* < 0.05).

## 3. Results

The operative surgical sites healed without incident. All of the implants were available for histological analysis. 

The mean insertion torque for the implants was 40.21 ± 0.87 N-cm in P2, 42.87 ± 0.11 N-cm in P3, and 44.68 ± 0.17 N-cm in P4. Using a paired two-sample *t*-test, a significant difference between the average insertion torques was found (*p* = 0.005) (Table 1).

The mean Implant Stability Quotient (ISQ) values were above 70 ISQ, which indicates a high primary stability, and they were increasing from day 0 to day 90. We can see, in Table 2 and Table 3, the ISQ values for wide ring implants and narrow ring implants.

The mean bone loss for the narrow ring implants is 0.75 ± 0.22 at 60 days and 0.89 ± 0.18 at 90 days in P2, 0.78 ± 0.19 at 60 days and 0.86 ± 0.59 at 90 days in P3, and 0.71 ± 0.11 at 60 days and 0.75 ± 0.11 at 90 days in P4, which indicates more bone loss at 90 days that at 60 days (Table 4).

The mean bone loss for the wide ring implants is 0.82 ± 0.11 at 60 days and 0.97 ± 0.91 at 90 days in P2, 0.80 ± 0.56 at 60 days and 0.89 ± 0.23 at 90 days in P3, and 0.79 ± 0.25 at 60 days and 0.79 ± 0.67 at 90 days in P4, which indicates more bone loss at 90 days that at 60 days (Table 5). In the microscopic analysis of the crestal bone remodeling, the distance from the implant shoulder to the first bone-to-implant contact was higher for the implants installed with a small ring in the buccal aspect with statistical significance (*p* < 0.001). For the peri-implant tissues thickness, the distance from the implant shoulder to the external portion of the epithelium showed no differences and no statistical significance was found in both types of implants.

## 4. Discussion

Short (length ≤ 8 mm) implants offer a minimally invasive alternative in the rehabilitation of atrophied alveolar bone [5].

Short implants present a similar success rate compared to conventional ones [14,29,30,31]. Those implants depend specifically on the cortical bone anchorage, because they are mainly used in highly resorbed areas, where the amount of cortical bone remains stable in comparison to the trabecular bone [32]. The main drawbacks of short implants are, on one hand, the lack of primary stability due to their small size [16], and the unfavorable crown-to-implant ratio [33,34]; therefore, adding elements to maximize the contact area and the mechanical retention in the dense cortical bone can be beneficial. In this experimental study in dogs, we tested a new short implant design in which a ring is added to the implant cervical area to improve the support and primary stability at the cortical bone level, in a similar way to the extraoral implants [35]. The addition of the ring would also prevent the implant from being inserted deeper than planned, which is very important when working next to delicate anatomical structures, such as the inferior alveolar nerve. The top of the ring is polished and the bottom has a rough surface, so it can become osseointegrated. To achieve a homogeneous seating of the ring on the bone crest, we used a round flattening reamer to achieve a flat surface, where the ring can rest homogenously.

Although a cervical ring can have some advantages from a mechanical point of view, it is important to test the biological behavior of this element, because the osseointegration of the bottom surface of the ring can increase the BIC area of the implant and improve the load transmission, but if the bone does not adhere to the rough bottom surface of the ring, the marginal bone loss will be increased and a higher incidence of peri-implantitis can be expected. No previous studies on the addition of such a ring on the osseointegration of this device have been published so far. There are very few animal studies on short implants [36,37], and they are in mandible and not in maxilla like this study. In 2016, our group published a pilot study with 60 extra-short 4 mm implants in a posterior mandible splinted with 10 mm length implants, with a 100% success rate at the one year follow up [38]. All of the implants of this study were correctly integrated, which is in line with the studies in humans that have a high success rate [39]. The perfect flattening of the bone crest is technically difficult and if the ring and the osteotomy are not perfectly aligned, the implant stops at the first bone contact. This fact explains why, when measuring the total values of the marginal bone loss, some higher values can appear. This would explain why the data have a lot of rank, and in the same implant there are areas with much more bone loss. If the measurements are made from the first bone implant, the contact the results will show different values. The latter is an important finding, because adding a circular element to the cervical area of a tissue level implant with a 2.0 mm neck is going to maintain the bone and can therefore provide a clinical benefit of a more primary and greater stability surface area of the implant in contact with the bone. More studies are needed with a smaller diameter ring that is more adapted to the animal´s jaw of the experiment, as well as the modification of the technique of insertion to be able to validate this assertion. Another issue is the long-term stability of the marginal bone in the ring area and the bone’s reaction to loading. Within the limitations of this study, the crestal bone resorption was reduced in the narrow extra-short ring implant designs, compared with wide ring implants in the healed maxilla. These data could be an important factor for humans, because the use of short implants with rings in soft and resorbed bone can be used with a high predictability, but can be managed with skillful technique.

More long term studies with loading protocols and different ring sizes must be performed. 

## 5. Conclusions

Our findings suggests that the amount of peri-implant tissues (crestal bone loss) after remodeling over a period of 12 weeks was smaller in the narrow ring extra short implant installed in the healed maxilla, compared with the wide ring extra-short implants.

## Figures and Tables

**Table 1:** Maximum insertion torque and median insertion torque of extra-short wide and narrow ring implants. SD—standard deviation.

Short DM^®^ Implant Position	Mean Maximum Insertion Torque (SD)	Median Insertion Torque	*p*-Value
P2	40.21 ± 0.87	40	0.824
P3	42.87 ± 0.11	42	0.456
P4	44.68 ± 0.17	44	0.012 *

**Table 2:** Implant Stability Quotient (ISQ) mean values at 0 days, 60 days, and at 90 days of the extra-short wide ring implants.

Short DM Implant Position	Mean (SD) ISQ Day 0	Median ISQ Day 0	Mean (SD) ISQ 60 Days	Median ISQ 60 Days	Mean (SD) ISQ 90 Days	Median ISQ 90 Days	*p*-Value
P2	72.23 ± 0.72	69.22–71.56	73.22 ± 0.34	72.70–77.16	74.29 ± 0.11	72.57–76.23	0.782
P3	76.56 ± 0.12	75.34–77.23	80.17 ± 0.62	79.37–83.28	80.56 ± 0.12	78.67–82.22	0.923
P4	78.33 ± 0.37	76.31–80.12	80.11 ± 0.39	78.14–83.12	82.34 ± 0.17	80.34–85.23	0.672

**Table 3:** ISQ mean values at 0 days, 60 days, and 90 days of the extra-short narrow ring implants.

Short DM Implant Position	Mean (SD) ISQ Day 0	Median ISQ Day 0	Mean (SD) ISQ 60 Days	Median ISQ 60 Days	Mean (SD) ISQ 90 Days	Median ISQ 90 Days	*p*-Value
P2	70.52 ± 0.41	69.81–72.76	73.45 ± 0.11	72.89–75.26	75.99 ± 0.76	74.38–78.33	0.782
P3	74.78 ± 0.11	73.22–76.18	78.66 ± 0.62	77.37–80.12	80.14 ± 0.89	78.67–82.78	0.923
P4	76.38 ± 0.22	74.11–78.11	79.81 ± 0.39	77.14–80.34	81.11 ± 0.34	80.34–83.14	0.672

**Table 4:** Bone loss at 60 days and 90 days of the extra-short narrow ring implant.

Time of Measurements	Mean (SD) Bone Loss at Short Implants P2 (mm)	Median Short Implants P2 (mm)	Mean (SD) Bone Loss at Short Implants P3 (mm)	Median at Short Implants P3 (mm)	Mean (SD) Bone Loss at Short Implants P4 (mm)	Median at Short Implants P4 (mm)	*p*-Value
60 days	0.75 ± 0.22	0.7	0.78 ± 0.19	0.7	0.71 ± 0.11	0.7	0.012 *
90 days	0.89 ± 0.18	0.8	0.86 ± 0.59	0.8	0.75 ± 0.52	0.7	0.134 *

**Table 5:** Bone loss at 60 days and 90 days of extra-short wide ring implant.

Time of Measurements	Mean (SD) Bone Loss at Short Implants P2 (mm)	Median Short Implants P2 (mm)	Mean (SD) Bone Loss at Short Implants P3 (mm)	Median at Short Implants P3 (mm)	Mean (SD) Bone Loss at Short Implants P4 (mm)	Median at Short Implants P4 (mm)	*p*-Value
60 days	0.82 ± 0.11	0.8	0.80 ± 0.56	0.8	0.79 ± 0.25	0.7	0.382
90 days	0.97 ± 0.91	0.9	0.89 ± 0.23	0.8	0.79 ± 0.67	0.7	0.572
